# Prevalence and Characteristics of Polyneuropathy in Atypical Parkinsonian Syndromes: An Explorative Study

**DOI:** 10.3390/brainsci11070879

**Published:** 2021-06-30

**Authors:** Rachel Rohmann, Eva Kühn, Raphael Scherbaum, Lovis Hilker, Saskia Kools, Leonard Scholz, Katharina Müller, Sophie Huckemann, Christiane Schneider-Gold, Ralf Gold, Kalliopi Pitarokoili, Lars Tönges, Eun Hae Kwon

**Affiliations:** 1 Department of Neurology, St. Josef-Hospital, Ruhr-University, D-44791 Bochum, Germany; Rachel.Rohmann@ruhr-uni-bochum.de (R.R.); eva.kuehn@rub.de (E.K.); raphael.scherbaum@rub.de (R.S.); lovis.hilker@rub.de (L.H.); saskia.kools@rub.de (S.K.); leonard.scholz@rub.de (L.S.); katharina.mueller@rub.de (K.M.); sophie.huckemann@rub.de (S.H.); Christiane.Schneider-Gold@ruhr-uni-bochum.de (C.S.-G.); ralf.gold@rub.de (R.G.); Kalliopi.Pitarokoili@ruhr-uni-bochum.de (K.P.); eunhae.kwon@rub.de (E.H.K.); 2 Neurodegeneration Research, Centre for Protein Diagnostics (ProDi), Ruhr-University, D-44791 Bochum, Germany

**Keywords:** polyneuropathy, atypical parkinsonian syndromes, multiple system atrophy, progressive supranuclear palsy, nerve conduction study

## Abstract

(1) Background: Peripheral nerve involvement is increasingly recognized in Parkinson’s disease (PD). Although non-motor symptoms and postural instability are early features of atypical parkinsonian syndromes (APS), peripheral neuropathies in APS have not been addressed in detail thus far. Therefore, the aim of this study was to investigate the prevalence and characteristics of polyneuropathies (PNP) in multiple system atrophy (MSA) and progressive supranuclear palsy (PSP), as representative syndromes of APS. (2) Methods: In total, 8 MSA and 6 PSP patients were comprehensively analyzed regarding subjective, clinical (motor and non-motor) and paraclinical PNP features using nerve conduction studies and high resolution nerve ultrasounds (HRUS). (3) Results: A total of 87.5% of MSA and 66.7% of PSP patients complained of at least one neuropathic symptom, with electrophysiological confirmation of PNP in 50.0% of both, MSA and PSP patients. PNP symptom severity in PSP and motor nerve amplitude in MSA were associated with compromised motor function. Morphologic nerve examination by HRUS showed few alterations according to the axonal type of PNP. (4) Conclusions: The overall high PNP symptom burden may be partially credited to the significant prevalence of electrophysiologically diagnosed PNP, and impact motor aspects of APS. The findings of this exploratory study reinforce further investigations on a larger scale, in order to elucidate peripheral nerve involvement and the underlying pathophysiological mechanisms of APS.

## 1. Introduction

Atypical parkinsonian syndromes (APS) are neurodegenerative disorders which present with additional features beyond parkinsonism, often associated with an insufficient response to levodopa and a more rapid disease course. Based on the pathogenic protein aggregates, multiple system atrophy (MSA) belongs to the spectrum of synucleinopathies alongside Parkinson’s disease (PD) and dementia with Lewy bodies (DLB), whereas progressive supranuclear palsy (PSP) and corticobasal degeneration (CBD) represent tauopathies. MSA is characterized by a variable combination of parkinsonism, cerebellar ataxia and autonomic failure, involving urinary incontinence, erectile dysfunction and orthostatic hypotension. The accumulation of alpha-synuclein in the cytoplasm of oligodendrocytes (glial cytoplasmic inclusions, GCI) in striatonigral and olivopontocerebellar structures presents as the pathological hallmark [1,2]. PSP manifests with multiple phenotypic variants, of which core symptoms consist of ocular motor dysfunction, postural instability, akinesia and cognitive impairment [3]. The clinical heterogeneity arises from the regional distribution of the 4-repeat tau protein deposition predominantly in the basal ganglia and brain stem [4].

Growing evidence on non-motor symptoms imply the complex multisystemic nature of APS. Non-motor symptoms, including pain, mood, cognition, sleep, gastrointestinal and urogenital disturbances are frequently prevalent and burdensome not only in PD, but also in APS [5,6]. While peripheral nerve involvement has been increasingly recognized in PD [7,8,9,10], only few studies have addressed peripheral nerve alterations in APS thus far. The prevalence of peripheral nerve abnormalities detected by nerve conduction studies is estimated up to 40% [11,12,13]. A causal link between alpha-synuclein and associated peripheral neuropathy remains unclear; however, alpha-synuclein aggregates are distributed throughout the peripheral nervous system, including sympathetic ganglia [14], skin nerve fibers [15] and Schwann cells [16]. Neurophysiological evidence of peripheral neuropathy in PSP is even more scarce [17]. However, cutaneous denervation involving small and large nerve fiber endings in PSP has recently been demonstrated [18].

In order to expand the knowledge on peripheral nerve involvement in APS, we aimed to analyze the frequency, clinical, electrophysiological and morphological characteristics of polyneuropathy (PNP) associated with MSA and PSP. Therefore, we applied the “Parkinson Nerve Study” design, which has been established in Parkinson’s disease [9], to explore the relevance of PNP in APS.

## 2. Materials and Methods

In this exploratory cross-sectional study we enrolled 8 MSA and 6 PSP patients at the Department of Neurology at St. Josef Hospital of Ruhr-University Bochum over the observation period from November 2018 to March 2020. All patients fulfilled the diagnostic criteria for at least possible MSA, according to the second consensus statement [1], and the MDS diagnostic criteria for PSP [3]. As we recruited well-characterized patients in our neurology clinic, medical history and essential laboratory results were accessible before screening and main exclusion criteria could be ruled out beforehand by prescreening. We assessed 10 MSA and 7 PSP patients for eligibility. Due to the presence of genetic alterations associated with parkinsonian syndromes, 2 MSA and 1 PSP patient were excluded. Main exclusion criteria were PNP-associated diseases, such as diabetes, alcohol abuse and other known causes of neuropathy. Written informed consent was obtained from all subjects before study initiation, and the study protocol was approved by the ethics committee of the Medical Faculty of the Ruhr University Bochum (register-number 18-6360, date of approval 9 December 2018). The study is listed in the German Clinical Trials Register (ID: DRKS00020752).

### 2.1. Clinical Assessments

The study procedures included a patient interview, neurological examination and modified Neuropathy Disability Score (NDS) [19,20]. Motor impairment was assessed using the Unified Multiple System Atrophy Rating Scale (UMSARS) [21], Progressive Supranuclear Palsy Rating Scale (PSPRS) [22] and MDS-Unified Parkinson’s Disease Rating Scale (MDS-UPDRS) [23]. To obtain a higher comparability among motor scores, we chose UMSARS part II (motor examination scale) and PSPRS part V+VI (limb exam and gait/midline exam) in analogy with UPDRS part III for analysis. We further assessed the MDS-UPDRS III parts individually in the 7 subdomains [23]. Non-motor symptoms were evaluated according to the Assessment of Autonomic Dysfunction in Parkinson Disease (SCOPA-AUT) [24], Non Motor Symptom Questionnaire (NMSQuest) [25], Montreal Cognitive Assessment (MoCA) [26], modified Neuropathy Symptom Score (NSS) [19,20] and Parkinson’s Disease Questionnaire (PDQ-39) [27]. To reflect the multidimensional character of the questionnaire, we analyzed the PDQ-39 summary index (PDQ-39 SI) as well as the 8 PDQ-39 subscales separately [28]. Due to individual reasons, the number of tested patients in MDS-UPDRS III, PSPRS, NDS, nerve conduction studies and HRUS examination may vary slightly from the total numbers in Table 1, but are indicated respectively.

### 2.2. Nerve Conduction Studies, HRUS and Diagnosis Criteria of PNP

Every patient underwent a motor examination of the median and tibial nerve, and a sensory examination of the median and sural nerve that were recorded bilaterally, using a Medtronic four channel electroneurography device (Medtronic, Meerbusch, Germany). Skin temperature was maintained at 36 °C during the procedure. To identify early PNP, the lower value of the bilateral nerve conduction was selected. On the basis of standard values [29], three PNP severity levels were determined (Table 2).

High-resolution ultrasound (HRUS) of peripheral nerves was conducted bilaterally at entrapment and non-entrapment sites. The cross-sectional area (CSA) of the nerves at entrapment sites was measured at the following locations: median nerve at carpal tunnel, ulnar nerve at Loge de Guyon and Sulcus ulnaris and fibular nerve at caput fibularis. The non-entrapment sites included median nerve at the upper arm, ulnar nerve at the upper arm and fibular nerve at fossa tibialis. All examinations were performed with the use of an Affinity^®^70 G ultrasound system (Philips, Hamburg, Germany), with an 18-MHz linear array transducer. By keeping the transducer perpendicular to the nerves, not applying additional force and maintaining the extremities in the neutral position, anisotropy and artificial nerve deformation could be prevented. The CSA values were measured at the inner border of the thin hyperechoic epineural rim by a continuous tracing technique [30].

### 2.3. Laboratory Assessment

In order to exclude other common causes of PNP, a broad laboratory screening was performed on all patients. The screening comprised of a blood cell count, HbA1c, liver enzymes, urea, creatinine, electrolytes, thyroid stimulating hormone, vitamin B12, B1, B6, methylmalonic acid, homocysteine, holotranscobalamin levels and serum protein electrophoresis/immunofixation.

### 2.4. Statistical Analysis

Statistical analysis was performed using SPSS version 25.0 (IBM Deutschland GmBH, Ehningen, Germany). If not otherwise indicated, clinical, demographic and electrophysiological data are shown as means with standard deviations (SD), and ordinal data are presented as median and interquartile range (IQR). The normal distribution of data was calculated using the Shapiro–Wilk test. Differences between two groups (e.g., demographical analysis of MSA and PSP patients subdivided into subgroups with/without electrophysiologically confirmed PNP) were analyzed by Student’s t-test if normally distributed, or Mann–Whitney U test if not normally distributed. Comparisons of categorial data were performed with Fisher’s exact test. To account for the possibility of error of the first kind, due to multiple testing, Bonferroni-Holm correction was used in post hoc analyses [31]. The findings, which failed to reach the significance level after Bonferroni–Holm correction, are indicated in the text. Median differences between PNP subgroups were assessed using the Hodges–Lehman estimate and are stated with the 95% Confidence Intervals. The Spearman correlation coefficient r was used for analysis between the electrophysiological results and clinical parameters. Differences were considered significant if *p* < 0.05. Considering the influence of age as a confounding factor of PNP, we performed partial correlations between peripheral nerve amplitudes and motor scores with a correction for age in the MSA group only, as assumptions regarding linearity and normal distribution were only partially fulfilled. 

## 3. Results

### 3.1. Demographic and Clinical Data

Table 1 summarizes the demographic and clinical characteristics of MSA and PSP patients, subdivided into groups with and without a electrophysiological diagnosis of PNP. In subgroup analysis, MSA patients with PNP showed a significantly higher mean levodopa equivalence dose (LED) (*p* = 0.048), which was not found to be significant after the Bonferroni–Holm Correction [*a* = 0.005] (Table 1).

### 3.2. Patient-Reported Neuropathy Symptoms and Functional Impact

**Figure 1:**
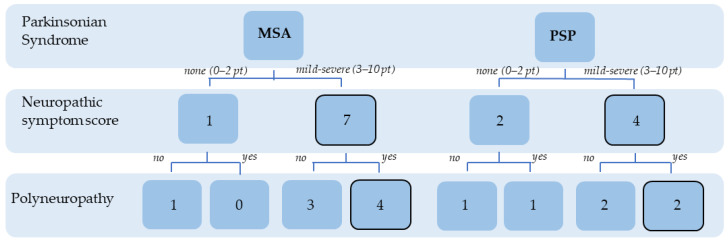
Prevalence of neuropathic symptoms in APS patients in the NSS and electrophysiologically confirmed PNP.

**Figure 2:**
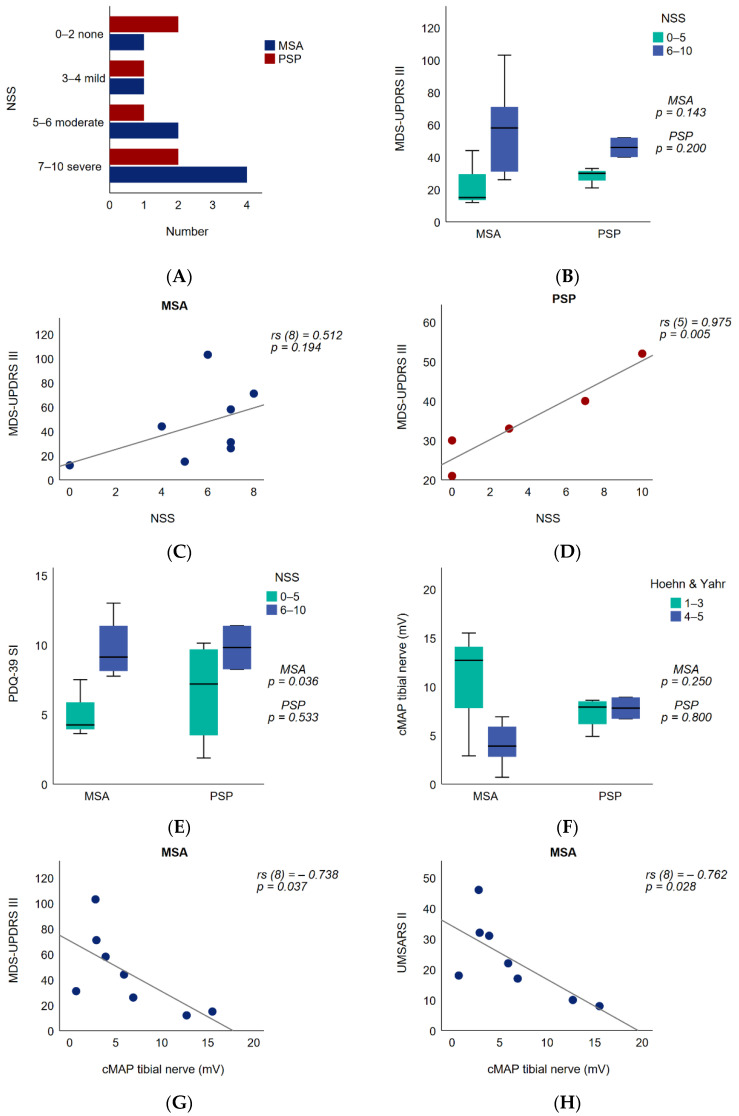
(**A**) NSS score. (**B**) MDS-UPDRS III in NSS groups. (**C**) MSA: MDS-UPDRS III in relation to NSS. (**D**) PSP: MDS-UPDRS III in relation to NSS. (**E**) PDQ-39 SI in NSS groups. (**F**) Amplitude of the tibial nerve in Hoehn and Yahr groups. (**G**) MSA: MDS-UPDRS III in relation to the amplitude of the tibial nerve. (**H**) MSA: UMSARS II in relation to the amplitude of the tibial nerve.

The NSS quantifies the subjective burden of PNP symptoms. In total, 87.5% of the MSA and 66.7% of the PSP patients presented at least one neuropathic phenomenon in the NSS. A total of four out of eight (50%) MSA patients fulfilled the electrodiagnostic criteria for PNP. One patient was categorized into a mild, sensory PNP. Two patients had a moderate, sensorimotor PNP, and one patient fulfilled the criteria for a severe, sensorimotor PNP. In PSP, three out of six (50%) patients fulfilled the electrodiagnostic criteria for PNP. Two patients were categorized into a mild, sensory PNP, and one patient fulfilled the criteria for a severe, sensorimotor PNP (Figure 1 and Figure 2A).

MSA patients with more severe neuropathic symptoms (NSS > 5 points) showed a significantly more severe compromised quality of life, as assessed by the PDQ-39 SI (*p* = 0.036), which was not significant after Bonferroni–Holm Correction [*a* = 0.005]) (Figure 2E, Table S1). Under consideration of the PDQ-39 subdomains, a significant effect was also found for the PDQ-39 subscale Mobility (*p* = 0.036), which was also not significant after Bonferroni–Holm Correction [*a* = 0.005]) (Table S1). In PSP patients, no association between groups of NSS and PDQ-39 SI was observed (*p* = 0.533) (Figure 2E, Table S1). In group comparison, no significant differences between the severity of NSS and motor scores could be demonstrated (Table S1).

The NSS value correlated significantly with the MDS-UPDRS III in PSP (Figure 2D), as well as with the MDS-UPDRS III factors midline function, bradykinesia of the right upper extremity and lower limb bradykinesia (MDS-UPDRS III: rs (5) = 0.975; *p* = 0.005) (Table S2). In MSA patients, we did not find significant associations between NSS severity and motor scores (Table S2).

### 3.3. Peripheral Nerve Motor Amplitude and Functional Impact

The tibial nerve cMAP amplitude in MSA and PSP patients with regard to the Hoehn and Yahr stage did not significantly differ in the subgroup analysis (MSA: *p* = 0.250; PSP: *p* = 0.800) (Figure 2F). In MSA patients, the tibial nerve cMAP amplitude showed a significant inverse correlation with the UMSARS II (r_s_ (8) = −0.762, *p* = 0.028; after age correction r (8) = −0.873; *p* = 0.010) (Figure 2H, Tables S2 and S3) and with the MDS-UPDRS III (r_s_ (8) = −0.738, *p* = 0.037; after age correction r (8) = −0.816; *p* = 0.025) (Figure 2G, Tables S2 and S3). Examining the MDS-UPDRS III subdomains, significant inverse relationships were found between the tibial nerve cMAP amplitude and the factors of midline function as well as postural and kinetic tremors (midline function: r_s_ (8) = −0.790, *p* = 0.020; postural and kinetic tremors: r_s_ (8) = −0.724, *p* = 0.042) (Tables S2 and S3). Interestingly, the significant findings remained after performing partial correlations with a correction for age, yielding even higher correlation coefficients (Table S3). In addition, the correlation between tibial nerve cMAP amplitude and the factors of bradykinesia of the upper extremity and lower limb bradykinesia proved to be significant after a correction for age (Table S3). No significant associations were demonstrated between the tibial nerve cMAP amplitude and motor scores in the PSP cohort (Table S2). Additionally, no statistically significant associations between the tibial nerve cMAP amplitude and age or disease duration were observed.

The group difference between the sural nerve sNAP amplitude and Hoehn and Yahr stage in MSA and PSP was not shown to be significant (MSA: *p* = 0.857; PSP: *p* = 0.267). The sural sNAP amplitude did not correlate significantly with the NSS or the motor scores in MSA or PSP patients (Table S2). There was no correlation between sural sNAP amplitude and age or disease duration. 

### 3.4. Nerve Alterations in High-Resolution Ultrasound of Peripheral Nerves

**Figure 3:**
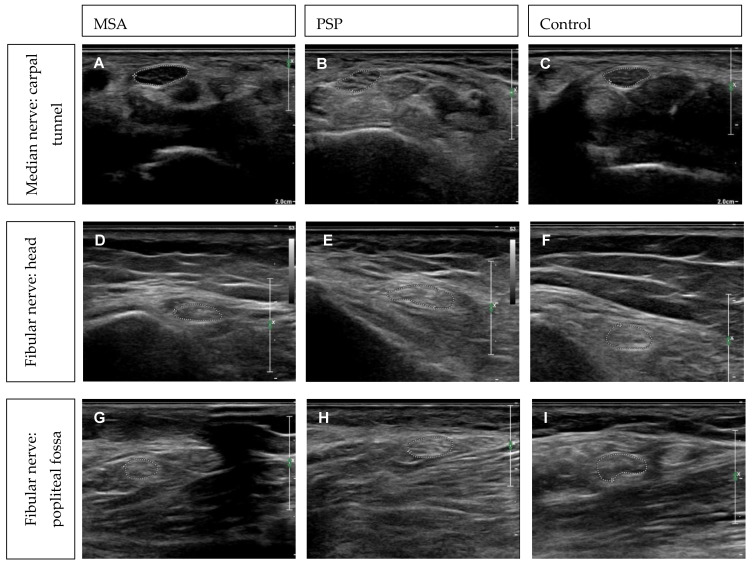
(**A**–**I**) High resolution ultrasound of peripheral nerves at entrapment sites of MSA (**A**,**D**,**G**) vs. PSP (**B**,**E**,**H**) vs. control. (**C**,**F**,**I**) (**A**–**C**) Median nerve: carpal tunnel. (**D**–**F**) Fibular nerve: head. (**G**–**I**) Fibular nerve: popliteal fossa.

To evaluate and visualize morphological nerve alterations, HRUS was applied on all patients at entrapment and non-entrapment sites of peripheral nerves, in relation to healthy control values [30]. The analysis of the entrapment sites showed enlarged cross-sectional-areas (CSA) of the median nerve at the carpal tunnel. This was larger than 12.57 mm^2^ in two out of five MSA patients and one out of six PSP patients. Three out of six MSA and three out of six PSP patients showed enlarged CSA (>7.22 mm^2^) of the ulnar nerve at the Loge de Guyon, and three out of six MSA and three out of six PSP patients demonstrated enlarged CSA (>8.13 mm^2^) values of the ulnar nerve in the ulnar sulcus. Regarding the entrapment site CSA of the fibular nerve at the fibular head, in three out of six MSA and in six out of six PSP patients enlarged results (>11.7 mm^2^) could be found (Figure 3).

Regarding non-entrapment sites, no morphological alterations based on the HRUS could be reported in the median nerve at the upper arm (>14.14 mm^2^) or the ulnar nerve at the upper arm (>10.17 mm^2^). One out of six MSA and four out of six PSP patients showed enlarged CSA of the fibular nerve in the popliteal fossa (>13.2 mm^2^) (Figure 3). No direct correlation between nerve conduction studies and HRUS alterations could be detected (Table 1).

### 3.5. Laboratory Assessment of PNP in APS

Other known common causes for PNP were excluded using a broad laboratory PNP panel (Table S4). Interestingly, we observed elevated levels of homocysteine (Hcy) and methylmalonate (MMA) in the MSA and PSP cohort, despite normal concentrations of vitamin B12 and folic acid (Table S4).

## 4. Discussion

In the present study, high levels of subjective neuropathic complaints in 87.5% of the MSA and 66.7% of the PSP patients were recorded by the patient self-report instrument NSS. Electrophysiological confirmation of PNP could be established in 50% of the MSA and 50% of the PSP patients, which considerably exceeds the rate of 7–9% in the normal elderly population [32,33], but is consistent with the reported PNP prevalence rates in PD, ranging from 16.3% up to 62% [9,34].

In comparison to previous nerve conduction studies in APS, the prevalence rate of PNP was higher in our study population. An earlier study reported a PNP rate of only 17% in 40 MSA patients, mainly presenting with a distal axonal sensorimotor neuropathy [11]. In another study, abnormal nerve conduction was found in 24% of 42 MSA patients [13]. Electrophysiological data were collected from a right leg that was arbitrarily chosen, whereas in our analysis, we selected the lower value of bilateral nerve conduction in order to detect early PNP, which could be accountable for the higher PNP rate in our study population. Moreover, our study group showed an older age at evaluation (61.1 ± 8.1 years in Abele et al.; 68.9 ± 9.2 years in our cohort) and at diagnosis (57.1 ± 8 years in Abele et al.; 65.5 ± 9.6 years in our cohort). The presence of PNP is strongly related to physiological aging, as the incidence of cryptogenic PNP increases with age [35]. However, we did not observe a significant age-dependent difference between patients with and without PNP. When we performed an age correction by partial correlation in the MSA group, we also found no significant age-dependent effect. In the nerve conduction study on the ulnar, peroneal and sural nerve of 48 MSA patients, axonal neuropathy in one nerve was found in 20.8%, and two nerves were affected in a further 20.8% [12]. Taking 41.8% ENG alterations in total, this PNP prevalence rate is closest to our own findings, including mild to severe PNP. EMG studies in MSA focused on the anal sphincter muscle that revealed signs of denervation and re-innervation in up to 90% of MSA patients [11]. Neurogenic EMG alterations in skeletal muscles were more frequently detected than nerve conduction abnormalities, pointing towards a neurodegeneration of the spinal anterior horn and Onuf’s nucleus in MSA [11,12].

The study group of Gawel et al. conducted one of the few electrophysiological examinations on 24 PSP patients [17]. The only alterations of the ulnar, peroneal and sural nerves could be detected in the form of reduced motor and sensory amplitudes in the ulnar nerve in 8.3% and 20% of the study population. Instead, neurogenic EMG abnormalities in skeletal muscles were present in almost half of the PSP patients, indicating lower motor neuron degeneration in the anterior horn, as supported by the neuropathological findings of a spinal cord involvement [36,37]. Interestingly, the EMG alterations showed a marginally significant correlation to the UPDRS part III score, suggesting that neuronal loss in the spinal cord may contribute to motor disability in PSP, although our electrophysiological findings did not correlate with motor scores in PSP patients. The skin biopsy analysis of 27 PSP patients demonstrated a length-dependent loss of sensory and autonomic nerve fibers associated with higher UPDRS part III scores [18]. Moreover, sensory nerve conduction revealed that sural nerve amplitudes were reduced further in the PSP group (mean 5.8 µV) compared to PD and controls, which remains higher than the mean sural amplitude of 2.7 µV in our PSP group.

In the “Parkinson Nerve Study” by Kühn et al., 86% of PD patients reported PNP symptoms, and a higher proportion of PD patients (62%) showed electrophysiological signs of PNP compared to our MSA and PSP population [9]. Although NSS is higher in PD, the NSS rate of MSA (87.5%) and PSP (66.7%) patients in our study with at least mild neuropathic symptoms (NSS > 2) remains high, compared to a diabetic population where only 48% have an NSS > 2 points [38]. In our study, PDQ-39-SI was found to be higher in MSA patients with a higher NSS, particularly with respect to the Mobility subscale, but this was not significant after Bonferroni–Holm Correction. In a Romanian cohort, the presence of PNP in PD patients had a negative impact on motor, activities of daily living, emotional well-being and body discomfort domains of health-related quality of life [39]. While NSS in PD patients significantly correlated with motor, non-motor symptoms as well as poor quality of life in our study, we found a significant correlation between NSS and UPDRS part III in PSP patients, including within the subscales of midline function and lower limb bradykinesia. Regarding electrophysiological alterations, Kühn et al. reported a correlation between motor nerve amplitudes in PD patients and motor impairment, disease duration and age [9]. Lower tibial cMAP amplitude in MSA patients in our study was associated with higher UMSARS II scores as well as UPDRS III scores, including midline function (facial expression, posture, gait) and lower limb bradykinesia. In addition to central mechanisms, PNP may increase functional motor disability through a loss of proprioception and delayed muscle responses contributing to postural instability [40]. Motor function of the lower body may also be impaired by PNP resulting in poor gait performance and bradykinetic movements.

In our study population, we observed a difference between the high prevalence of patient-reported PNP phenomena and the less frequent electrophysiological confirmation of PNP in the MSA and PSP group. Thus, PNP symptoms in APS may also be attributable to a central origin. Non-motor symptoms, including autonomic dysfunction and pain dominate the clinical picture of APS [6,41]. The facilitation of nociceptive processing at the spinal level, as demonstrated in advanced PSP patients, as well as the marked involvement of non-dopaminergic central sensory pathways could enhance subjective neuropathic symptom perception in APS [42]. Another aspect to consider in this context is that the small fiber neuropathies that contribute to sensory and mechanoreceptive disturbances are not evaluated by nerve conduction studies. The lower subjective perception of PNP symptoms in PSP patients with similar PNP rates compared to MSA patients may be related to neurocognitive deficits with the reduced awareness of somatic symptoms (“loss of insight”) [43]. Additional factors, such as the neurotoxic influence from levodopa and metabolites (Hcy, MMA), have involved controversial discussion, as they cause neuropathic changes [44]. The levodopa dosage did not differ significantly in MSA patients with and without PNP. The elevated levels of Hyc and MMA in the MSA and PSP patients of our study are in line with findings that suggest that these metabolites are a consequence of, or surrogate marker for neurodegeneration [30].

As PNP characteristics of MSA resemble that of PD more than of PSP, underlying pathogenetic differences in synucleinopathies vs. tauopathies are implied. Early histological sural biopsies of MSA patients revealed a loss of myelinated and unmyelinated fibers [45,46,47]. Given the multisystemic nature of MSA, peripheral alpha-synuclein pathology is widespread, and was detected in the sympathetic ganglia, cranial, spinal and autonomic nerves in MSA patients [14,16]. Skin biopsies revealed alpha-synuclein deposits only in MSA and PD patients and not in tauopathies, as MSA patients showed mainly unmyelinated somatosensory fibers [15]. Deposition of alpha-synuclein was also found in the cytoplasm of Schwann cells [16,48]. The way in which alpha-synuclein interacts with neuronal axon and myelin is unclear; however, it has been discussed that the overexpression of alpha-synuclein in Schwann cells may impair the activity of neurotrophic factors, leading to axonal destabilization in peripheral nerves [16,48]. As for PSP, evidence of tau-pathology in the peripheral nerves in PSP is limited and inconclusive. Neurofibrillary tangles were observed in the spinal ganglia of PSP patients [49], but this could not be replicated in a further immunohistochemical study [50], Furthermore, the skin tissue of PSP patients displayed a higher tau-immunoreactivity compared to those of controls [51]. The finding of cutaneous denervation in PSP should encourage to further investigation of small fiber neuropathy in PSP as well as MSA [18].

For the first time, HRUS examination was performed on APS patients. We could detect only few morphologic alterations with increased CSA in MSA and PSP patients at entrapment (carpal tunnel, sulcus ulnaris, fibular head) and non-entrapment sites (fossa tibialis). Such alterations differ from demyelinating neuropathies, but have been reported in other axonal types of neuropathies such as diabetes and restless-legs-syndrome [52,53]. In PD patients, morphological alterations occurred at entrapment sites, likely due to mechanical irritation and increased nerve vulnerability [9]. HRUS expands the characterization of PNP in a non-invasive manner and requires further evaluation, particularly from a longitudinal perspective.

The major limiting factor of our monocentric study is the small number of patients that does not allow a complex statistical analysis. The study data represent only a cross-sectional evaluation. Longitudinal follow-up examinations are needed to assess the temporal dynamics of PNP, alongside the motor and non-motor symptoms of the parkinsonian syndrome. However, this first analysis was conceptualized as an explorative study, with the objective to identify the relevance of PNP in APS based on the protocol of the “Parkinson Nerve Study” in PD. In future, this will be expanded to a larger number of patients in a longitudinal design.

## 5. Conclusions

In summary, we observed a high presence of subjective PNP burden in APS, particularly in MSA patients, that may be at least partially credited to the considerable prevalence of electrophysiologically diagnosed peripheral neuropathy. The severity of PNP symptoms impacted motor function in PSP patients. In MSA patients, the electrophysiological parameters were associated with worse motor scores, implicating that peripheral nerve impairment further impacts motor deficits in APS. HRUS is a helpful additional tool for the detection and morphologic characterization of PNP that requires further evaluated in APS. Our findings of the high prevalence and functional relevance of PNP in APS reinforce further investigations on a larger scale, including EMG, nerve and skin biopsies, to better understand the pathophysiological mechanisms of peripheral neurodegeneration in APS.

## Figures and Tables

**Table 1:** Demographical, clinical and instrument-based analysis of MSA and PSP patients, subdivided into groups with/without electrophysiologically confirmed polyneuropathy, and findings of high-resolution ultrasound examination at entrapment and non-entrapment sites.

	Total MSA Patients	MSA Patients without PNP (A)	MSA Patients with PNP (B)	Median Difference	95% CI		*p* Value
	(n = 8)	(n = 4)	(n = 4)		LL	UL	(A vs. B)
Age at evaluation (years)	68.9 ± 9.2	63.3 ± 8.2	74.5 ± 6.8	−11.0	−25.0	2.0	0.078
Female	4	1	3	n.a.	n.a.	n.a.	0.486
Disease duration (years)	3.3 ± 2.3	2.3 ± 1.5	4.3 ± 2.6	−1.5	−7.0	1.0	0.235
Age at diagnosis (years)	65.5 ± 9.6	60.8 ± 9.1	70.3 ± 8.5	−9.5	−24.0	9.0	0.176
Hoehn & Yahr, Median (IQR)	4 ± 1.4	3.3 ± 2.6	4 ± 0.8	−0.3	−2.0	1.0	0.686
Levodopa equivalence dose	705 ± 327.7	486.8 ± 207.3	923.3 ± 283.9	−464.5	−928.0	−62.0	0.048
MDS-UPDRS III Score	45 ± 31.1	39 ± 43.1	51 ± 17.3	−24.0	−56.0	59.0	0.624
UMSARS II Score	23 ± 12.7	20.3 ± 17.6	25.8 ± 6.8	−11.0	−23.0	24.0	0.581
NSS Score	5.5 ± 2.6	4.5 ± 3.1	6.5 ± 1.7	−1.5	−7.0	2.0	0.304
NDS Score ^1^	2.3 ± 1.8	1.5 ± 1.9	3.3 ± 1.2	−2.0	−4.0	2.0	0.206
PDQ-39 SI	8.1 ± 3.2	7.5 ± 4.4	8.7 ± 1.8	−2.8	−7.1	5.3	0.636
**NCS: Amplitude (mV)**							
N. suralis ^2^	2.8 ± 2.9	4 ± 3.5	1.9 ± 2.5	2.2	−5.2	6.7	0.393
N. tibialis	6.4 ± 5.2	9.5 ± 5.7	3.4 ± 2.2	6.5	−1.1	12.6	0.118
N. medianus (motor) ^1^	6.1 ± 3.4	6.3 ± 3.2	5.8 ± 4.3	0.7	−6.3	9.1	0.875
N. medianus (sensory) ^1^	6.8 ± 4.9	9.1 ± 4.8	3.7 ± 3.6	4.5	−1.8	16.0	0.168
**HRUS: CSA of Entrapment Sites (mm** ^2^ **)**							
N. medianus (Carpal tunnel) ^3^	10.9 ± 2	11 ± 2.3	10.7 ± 0	0.3	−2.3	3.2	0.900
N. fibularis (Caput fibularis) ^4^	13.4 ± 5.9	11.3 ± 1.6	17.6 ± 10.7	−6.2	−15.8	3.4	0.800
**HRUS: CSA of Non-Entrapment Sites (mm** ^2^ **)**							
N. medianus (upper arm) ^4^	9 ± 1.3	9.3 ± 1.3	8.2 ± 1.3	1.4	−0.5	4.1	0.385
N. fibularis (fossa tibialis) ^4^	9.2 ± 3.8	7.4 ± 1.5	12.8 ± 5.1	−5.4	−10.7	−0.1	0.370
	**Total PSP Patients**	**PSP Patients without PNP (C)**	**PSP Patients with PNP (D)**	**Median Difference**	**95% CI**		***p* Value**
	**(n = 6)**	**(n = 3)**	**(n = 3)**		**LL**	**UL**	**(C vs. D)**
	M ± SD	M ± SD	M ± SD				
Age at evaluation (years)	72.7 ± 10.2	74 ± 5.2	71.3 ± 15	5.0	−18.0	21.0	0.786
Female	1	0	1	n.a.	n.a.	n.a.	1.000
Disease duration (years)	4.5 ± 3.4	3.3 ± 1.5	5.7 ± 4.7	−1.0	−9.0	3.0	0.700
Age at diagnosis (years)	67.7 ± 13.6	70.3 ± 7.2	65 ± 19.7	6.0	−21.0	31.0	0.682
Hoehn & Yahr, Median (IQR)	3 ± 1	3 ± 0	3 ± 0	0.0	−1.0	1.0	1.000
Levodopa equivalence dose	410.8 ± 392.9	475 ± 532.1	346.7 ± 300.9	0.0	−540.0	1050.0	0.735
MDS−UPDRS III Score ^5^	35.2 ± 11.6	36.5 ± 21.9	34.3 ± 5.1	1.5	−19.0	22.0	0.912
PSPRS V + VI Score ^6^	14 ± 4.7	14.5 ± 6.4	13.5 ± 4.9	1.0	−7.0	9.0	0.877
NSS Score	4.2 ± 4	5 ± 5	3.3 ± 3.5	2.0	−7.0	10.0	0.661
NDS Score	4 ± 1.8	4.7 ± 2.3	3.3 ± 1.2	2.0	−2.0	4.0	0.422
PDQ-39 SI	7.7 ± 3.5	8.6 ± 3.2	6.8 ± 4.3	1.3	−5.0	9.5	0.586
**NCS: Amplitude (mV)**							
N. suralis	2.7 ± 2.2	4.2 ± 1	1.2 ± 2.1	3.3	−0.4	5.2	0.096
N. tibialis	7.5 ± 1.5	8.3 ± 0.8	6.7 ± 1.8	1.9	−1.0	4.0	0.215
N. medianus (motor)	5.4 ± 2.2	6.6 ± 2.5	4.1 ± 0.5	3.6	−0.8	4.8	0.400
N. medianus (sensory)	7.2 ± 4.8	6.3 ± 2.4	8 ± 7	−4.4	−9.2	8.7	0.707
**HRUS: CSA of Entrapment Sites (mm** ^2^ **)**							
N. medianus (Carpal tunnel)	9 ± 2.1	10.4 ± 2	7.7 ± 0.9	2.4	0.7	6.1	0.102
N. fibularis (Caput fibularis)	19.1 ± 9.1	16.7 ± 6.7	21.5 ± 12.1	−2.6	−23.6	10.0	0.400
**HRUS: CSA of Non-Entrapment Sites (mm** ^2^ **)**							
N. medianus (upper arm)	8.9 ± 1.4	9.4 ± 1.1	8.4 ± 1.7	0.8	−1.6	3.9	0.425
N. fibularis (fossa tibialis)	11.3 ± 3.8	10 ± 5.4	12.6 ± 1.5	−4.4	−7.2	5.3	0.466

^1^ n = 7, without PNP = 4, with PNP = 3; ^2^ n = 7, without PNP = 3, with PNP = 4; ^3^ n = 5, without PNP = 4, with PNP = 1; ^4^ n = 6, without PNP = 4, with PNP = 2. ^5^ n = 5, without PNP = 2, with PNP = 3; ^6^ n = 4, without PNP = 2, with PNP = 2. Abbreviations: CSA, cross-sectional area; MDS-UPDRS, MDS-Unified Parkinson’s Disease Rating Scale; UMSARS, Unified Multiple System Atrophy Rating Scale; PSPRS, Progressive Supranuclear Palsy Rating Scale; NSS, Neuropathy Symptom Score; NDS, Neuropathy Disability Score; PDQ-39, Parkinson’s Disease Questionnaire.

**Table 2:** Electrophysiological classification for PNP severity.

mild, sensory PNP	amplitude of the sural sNAP <5 mV for patients <50 years or <3.6 mV for patients >50 years amplitude of the tibial cMAP >5 mV amplitude of the median cMAP >5 mV
moderate, sensorimotor PNP	amplitude of the sural sNAP <5 mV for patients <50 years and <3.6 mV for patients >50 years amplitude of the tibial cMAP <5 mV amplitude of the median cMAP >5 mV
severe, sensorimotor PNP	amplitude of the sural sNAP <5 mV for patients <50 years and <3.6 mV for patients >50 years amplitude of the tibial cMAP <5 mV amplitude of the median cMAP <5 mV

## Data Availability

The data are not publicly available due to the privacy concern raised by our IRB.

## Supplementary Materials

The following are available online at https://www.mdpi.com/article/10.3390/brainsci11070879/s1, Table S1: Analysis of motor scores and PDQ-39 in MSA and PSP patients divided into subgroups of NSS 0–5 / 6–10, Table S2: Bivariate correlation analyses between nerve conduction studies, NSS and motor scores in MSA and PSP patients, Table S3: Partial correlation analyses between nerve conduction studies and motor scores in MSA patients after controlling for confounding variable age, Table S4: Laboratory parameters subdivided into subgroups with/without electrophysiologically confirmed polyneuropathy.
