# Supplementary material for: Prevalence and Characteristics of Polyneuropathy in Atypical Parkinsonian Syndromes: An Explorative Study
Source: Brain Sci. 2021 Jun 30;11(7):879. doi: 10.3390/brainsci11070879 (PMC8301815; doi:10.3390/brainsci11070879)
Supplement: Supplementary file 1 [file brainsci-11-00879-s001.zip › brainsci-1255611-supplementary.pdf]

Table S1. Analysis of motor scores and PDQ-39 in MSA and PSP patients divided into subgroups of NSS 0-5 / 6-10.

	**MSA**			**PSP**		
	**NSS 0-5 (n = 3)**	**NSS 6-10 (n = 5)**	**p value**	**NSS 0-5 (n = 4)**	**NSS 6-10 (n = 2)**	**p value**
	M ± SD	M ± SD		M ± SD	M ± SD	
UMSARS II	13.3 ± 7.6	28.8 ± 11.9	0.143	n.a.	n.a.	n.a.
PSPRS V+VI [*1]	n.a.	n.a.	n.a.	10 ± 0	18 ± 1.4	0.333
MDS-UPDRS III [*2]	23.7 ± 17.7	57.8 ± 31.4	0.143	28 ± 6.2	46 ± 8.5	0.200
Factor 1 Midline function [*2]	6 ± 7.8	19 ± 7.1	0.071	9 ± 2.6	18.5 ± 2.1	0.200
Factor 2 Rest tremor [*2]	0 ± 0	1.4 ± 3.1	0.786	0 ± 0	0 ± 0	1.000
Factor 3 Rigidity [*2]	6.7 ± 4.2	10 ± 7.4	0.786	5 ± 1	5 ± 1.4	1.000
Factor 4 Bradykinesia right UEX [*2]	4.7 ± 2.1	6.6 ± 3.6	0.571	4.3 ± 1.2	8 ± 1.4	0.200
Factor 5 Bradykinesia left UEX [*2]	2.3 ± 2.1	7.6 ± 4	0.143	3.7 ± 1.2	7.5 ± 2.1	0.200
Factor 6 Postural and kinetic tremors [*2]	1 ± 1.7	4 ± 4.7	0.250	1.3 ± 1.5	0 ± 0	0.400
Factor 7 Lower limb bradykinesia [*2]	3 ± 3.6	9.2 ± 4.3	0.143	4.7 ± 1.2	7 ± 1.4	0.200
PDQ-39 SI	5.1 ± 2.1	9.9 ± 2.2	**0.036**	6.6 ± 3.8	9.8 ± 2.2	0.533
PDQ-39 1 Mobility	21.7 ± 27	80 ± 16	**0.036**	48.1 ± 31	62.5 ± 14.1	0.800
PDQ-39 2 Activities of daily living	26.4 ± 19.7	61.7 ± 26.4	0.143	38.5 ± 20.5	56.3 ± 14.7	0.533
PDQ-39 3 Emotional well-being	27.8 ± 17.3	48.3 ± 9.6	0.071	24 ± 28.7	41.7 ± 35.4	0.533
PDQ-39 4 Stigma	31.3 ± 31.3	38.8 ± 30.1	0.786	28.1 ± 30	46.9 ± 66.3	0.800
PDQ-39 5 Social support	19.4 ± 33.7	18.3 ± 22.4	1.000	14.6 ± 18.5	4.2 ± 5.9	0.533
PDQ-39 6 Cognitions	33.3 ± 21.9	27.5 ± 12.2	0.786	35.9 ± 28.1	31.3 ± 35.4	0.800
PDQ-39 7 Communication	25 ± 14.4	36.7 ± 25.4	0.393	41.7 ± 11.8	75 ± 23.6	0.133
PDQ-39 8 Bodily discomfort	38.9 ± 31.5	36.7 ± 24	0.786	18.8 ± 18.5	70.8 ± 5.9	0.133

[*1] n for PSP = 4, NSS 0-5 n = 2, NSS 6-10 n = 2; [*2] n for PSP = 5, NSS 0-5 n = 3, NSS 6-10 n = 2

Table S2. Bivariate correlation analyses between nerve conduction studies, NSS and motor scores in MSA and PSP patients.

	**MSA**						**PSP**					
	**sNAP sural nerve (μV) (n = 7)**		**cMAP tibial nerve (mV) (n = 8)**		**NSS (n = 8)**		**sNAP sural nerve (μV) (n = 6)**		**cMAP tibial nerve (mV) (n = 6)**		**NSS (n = 6)**	
	r_s	p	r_s	p	r_s	p	r_s	p	r_s	p	r_s	p
NSS	-0.738	0.058	-0.561	0.148	n.a.	n.a.	-0.353	0.493	-0.232	0.658	n.a.	n.a
UMSARS II	-0.299	0.514	-0.762	**0.028**	0.464	0.247	n.a.	n.a.	n.a.	n.a.	n.a.	n.a
PSPRS V+VI [*1]	n.a.	n.a.	n.a.	n.a.	n.a.	n.a.	-0.056	0.944	-0.738	0.262	0.949	0.051
MDS-UPDRS III [*2]	-0.299	0.514	-0.738	**0.037**	0.512	0.194	-0.667	0.219	-0.5	**0.391**	0.975	**0.005**
Factor 1 Midline function [*2]	-0.274	0.552	-0.79	**0.020**	0.565	0.145	-0.667	0.219	-0.5	**0.391**	0.975	**0.005**
Factor 2 Rest tremor [*2]	0.321	0.483	-0.412	0.310	-0.085	0.842	0.000	0.000	0.000	0.000	0.000	0.000
Factor 3 Rigidity [*2]	-0.359	0.429	-0.192	0.649	0.221	0.599	0.081	0.897	-0.158	0.800	0.162	0.794
Factor 4 Bradykinesia right UEX [*2]	-0.048	0.919	-0.615	0.105	0.161	0.704	-0.553	0.334	-0.616	0.269	0.921	**0.026**
Factor 5 Bradykinesia left UEX [*2]	-0.449	0.312	-0.452	0.260	0.390	0.339	-0.237	0.701	-0.667	0.219	0.763	0.133
Factor 6 Postural and kinetic tremors [*2]	-0.171	0.713	-0.724	**0.042**	0.352	0.392	-0.057	0.927	0.783	**0.118**	-0.344	0.571
Factor 7 Lower limb bradykinesia [*2]	-0.500	0.253	-0.683	0.062	0.515	0.191	-0.649	0.236	-0.211	0.734	0.973	**0.005**
PDQ-39 Score	-0.318	0.487	-0.643	0.086	0.561	0.148	-0.435	0.389	0.086	0.872	0.464	0.354
PDQ-39 1 Mobility	-0.318	0.487	-0.667	0.071	0.512	0.194	-0.319	0.538	-0.029	0.957	0.087	0.870
PDQ-39 2 Activities of daily living	0.187	0.688	-0.595	0.120	0.171	0.686	-0.559	0.249	-0.319	0.538	0.132	0.803
PDQ-39 3 Emotional well-being	-0.019	0.968	-0.826	**0.011**	0.565	0.145	-0.551	0.257	-0.143	**0.787**	0.319	0.538
PDQ-39 4 Stigma	-0.255	0.581	-0.168	0.691	0.061	0.885	-0.103	0.846	0.406	0.425	0.309	0.551
PDQ-39 5 Social support	-0.155	0.739	-0.254	0.544	0.000	1.000	-0.329	0.525	0.062	0.908	-0.376	0.463
PDQ-39 6 Cognitions	-0.892	**0.007**	0.356	0.387	0.340	0.410	0.235	**0.654**	0.493	0.321	-0.221	0.674
PDQ-39 7 Communication	-0.397	0.379	0.048	0.910	0.173	0.682	0.000	1.000	-0.265	0.612	0.851	**0.032**
PDQ-39 8 Bodily discomfort	0.255	0.581	0.371	0.365	-0.270	0.518	-0.261	0.618	-0.257	0.623	0.696	0.125

[*1] n for PSP = 4; [*2] n for PSP = 5

Table S3. Partial correlation analyses between nerve conduction studies and motor scores in MSA patients after controlling for confounding variable age.

	MSA			
	sNAP sural nerve (µV) (n = 7)		cMAP tibial nerve (mV) (n = 8)	
	r_s	p	r_s	p
NSS	-0.593	0.215	-0.163	0.727
UMSARS II	-0.061	0.908	**-0.873**	**0.010**
MDS-UPDRS III	-0.036	0.946	**-0.816**	**0.025**
Factor 1 Midline function	-0.060	0.910	**-0.891**	**0.007**
Factor 2 Rest tremor	0.365	0.476	-0.698	0.081
Factor 3 Rigidity	-0.053	0.921	-0.384	0.395
Factor 4 Bradykinesia right UEX	0.054	0.919	**-0.771**	**0.043**
Factor 5 Bradykinesia left UEX	-0.281	0.590	**-0.828**	**0.021**
Factor 6 Postural and kinetic tremors	0.203	0.699	**-0.800**	**0.031**
Factor 7 Lower limb bradykinesia	-0.345	0.503	**-0.846**	**0.016**

Table S4. Laboratory parameters subdivided into subgroups with/without electrophysiologically confirmed polyneuropathy.

		Total MSA patients (n=8)	MSA patients without PNP (n=4)	MSA patients with PNP (n=4)	Total PSP patients (n=6)	PSP patients without PNP (n=3)	PSP patients with PNP (n=3)
**Parameter**	**Unit**	**M ± SD**	**M ± SD**	**M ± SD**	**M ± SD**	**M ± SD**	**M ± SD**
Leukocytes	/µl	7326.3 ± 1499.8	6920 ± 1317.4	7732.5 ± 1753	6635 ± 1179.4	6350 ± 1499.6	6920 ± 992.4
Erythrocytes	Mio/µl	4.8 ± 0.3	4.9 ± 0.2	4.6 ± 0.3	4.7 ± 0.5	4.4 ± 0.3	4.9 ± 0.5
HbA1c	%	5.5 ± 0.3	5.5 ± 0.3	5.6 ± 0.2	5.7 ± 0.3	5.8 ± 0.2	5.5 ± 0.5
GOT	µ/L	20.6 ± 3.6	18.8 ± 2.1	22.5 ± 4	22.3 ± 3.8	22.7 ± 3.8	22 ± 4.6
GPT	µ/L	16.5 ± 7.3	20.3 ± 5.7	12.8 ± 7.3	18.5 ± 7.1	15 ± 3	22 ± 9
GGT	µ/L	21.3 ± 6.1	24.5 ± 4.9	18 ± 5.8	44.8 ± 44	27.3 ± 4.2	62.3 ± 62.5
Urea	mg/dl	34.1 ± 11.8	32 ± 8.1	36.3 ± 15.6	33.8 ± 14.6	26.3 ± 10.8	41.3 ± 15.6
Creatinine	mg/dl	0.8 ± 0.1	0.9 ± 0.1	0.7 ± 0	1 ± 0.4	0.9 ± 0.2	1.2 ± 0.5
Sodium	mmol/l	140.3 ± 7.2	143.3 ± 2.1	137.3 ± 9.7	141.2 ± 1.2	141.7 ± 0.6	140.7 ± 1.5
Potassium	mmol/l	4.1 ± 0.5	4.4 ± 0.1	3.7 ± 0.6	4.2 ± 0.4	4.4 ± 0.1	4 ± 0.5
TSH	µIE/ml	1.6 ± 1.2	1.6 ± 1.7	1.6 ± 0.7	1.2 ± 0.4	1.2 ± 0.6	1.2 ± 0.3
Vitamin B12	pg/ml	592.4 ± 368.5	418.3 ± 125.5	766.5 ± 469.3	378.5 ± 81.5	327.7 ± 27.4	429.3 ± 90.1
Folic acid	ng/ml	8.3 ± 3.4	6.3 ± 2.4	10.3 ± 3.1	8.1 ± 5.9	6.1 ± 0.5	10.2 ± 8.6
Vitamin B1	µg/l	53.6 ± 12.9	52.8 ± 16.8	54.4 ± 10.1	60.1 ± 28	47.5 ± 14.8	72.6 ± 35.7
Vitamin B6	µg/l	12.2 ± 7.7	10.5 ± 5.6	13.9 ± 10	13 ± 5	13.3 ± 5.1	12.6 ± 5.9
Methylmalonic acid [*1]	nmol/l	303.9 ± 110.1	333.9 ± 152.2	274 ± 66.7	298 ± 98.2	346 ± 123.9	250.1 ± 43.1
Homocysteine [*2]	µmol/l	19.3 ± 3.7	20.9 ± 3.2	16 ± 2.1	20.3 ± 7.2	19.8 ± 5.5	20.8 ± 9.9
Holotranscobalamine	pmol/l	120.8 ± 48	126 ± 29.7	115.6 ± 66.5	75.5 ± 32.8	51.8 ± 6.2	99.2 ± 31

[*1] n for MSA = 6, without PNP = 3, with PNP = 3; [*2] n for MSA = 6, without PNP = 4, with PNP = 2
